# Opinions and Treatment Decisions for Dental Erosive Wear: A Questionnaire Survey among Icelandic Dentists

**DOI:** 10.1155/2018/8572371

**Published:** 2018-11-01

**Authors:** Aida Mulic, Inga B. Árnadòttir, Torbjòrg Jensdottir, Simen E. Kopperud

**Affiliations:** ^1^Nordic Institute of Dental Materials (NIOM), Oslo, Norway; ^2^Faculty of Odontology, University of Iceland, Reykjavík, Iceland; ^3^IceMedico ehf, Reykjavìk, Iceland; ^4^Specialist Oral Health Centre for Western Norway, Stavanger, Norway

## Abstract

Dental erosive wear (DEW) is common among children and adolescents, and a survey of Icelandic children showed that 30.7% of 15-year-olds were diagnosed with the condition.* Objective.* To gain knowledge about dental practitioners' experiences, opinions, and treatment decisions. *Materials and Methods*. A precoded questionnaire, previously used among Norwegian dentists, was sent electronically to all dentists in Iceland (*n* = 341). *Results.* The response rate was 64.2%, and 58% of dentists were male. More than half of the clinicians (54%) thought that prevalence had increased the last 10–15 years, and 67% reported it to be more common in male. Most (96%) recorded presence of DEW, but only 4% used a detailed scoring system. Lesions were mostly on occlusal surfaces of first mandibular molars (73%), on palatal in upper anterior teeth (61%), and on occlusal of maxillary first molars (36%). Most dentists (74%) reported a probable cause, e.g., high consumption of carbonated beverages (98%), acidic juices (68%), sport drinks (58%), reflux (54%), and eating disorders (20%). Dietary history was often recorded by 38%, and 65% never measured saliva. Most of the dentists (88%) treated patients themselves, and half of them preferred prevention with high fluoride and resin sealants. While some dentists wanted to restore teeth more invasively, most considered to restore with a filling. *Conclusion*. Icelandic dentists seem to be well educated for diagnosis and treatment of dental erosion, and dentists are aware of a minimally invasive approach. *Clinical Significance.* It is challenging for dentists to make the best treatment decision for patients with DEW, both in a short perspective and long perspective. At present, little is known about their knowledge and treatment approach, and there is no standard treatment which can be recommended. Therefore, the present study investigated dental practitioners' treatment decisions, as well as knowledge, experiences, and awareness of DEW.

## 1. Introduction

During recent decades, there has been an increased focus and interest on dental erosive wear in children, adolescents, and young adults [1]. Several prevalence studies have been performed, with estimated prevalence in permanent teeth reported to be high—on an average of 30% worldwide [2]. Prevalence studies in the Nordic countries confirm a high and increasing prevalence of dental erosions [3–11]. The earliest of these studies from 2010 among a cohort of 20% of all 15-year-olds in Reykjavík found the prevalence to be 21.6%, while 30.7% of the 15-year-olds had dental erosive lesions [11]. Dentine lesions were found in 5.5% of these. Relatively similar distribution has also been confirmed among Norwegian 16- to 18-year-olds, with a prevalence varying from 32% to 64% [7–9].

The high prevalence, often related to a high severity, is considered as a challenge, not only to the patients, but also to the dental health professionals. The foremost important task for both dental health personnel and patients is to identify and to limit, or eliminate, the acid exposures. However, for some patients, that will not be sufficient, and additional operative treatment of the lesions may be required. It is challenging for dental clinicians to make the best treatment decision for these, often young patients, both in a short and long perspective. At present, there is no standard treatment that can be recommended for teeth with dental erosive wear. However, a minimally invasive approach has been encouraged [12].

Up till now, little is known about dental health professionals' knowledge and treatment approach related to dental erosive wear. In Norway, a questionnaire study was performed among dentists in 2011, recognizing varying approach related to identifying, knowledge, and treatment of the condition [13]. However, no similar studies have yet been performed in other Nordic countries. Therefore, the aim of the present study was to gain knowledge about Icelandic dental practitioners' experiences, awareness, and knowledge of dental erosive wear. In addition, we aimed to investigate dentists' treatment decisions of teeth affected with dental erosive wear, illustrated by two specific patient cases with different severity.

## 2. Materials and Methods

A precoded questionnaire was sent electronically to all dentists (*n*=341) in Iceland in September 2016, using the Internet-based software QuestBack (Oslo, Norway). The questionnaire software was configured to automatically send up to four reminders to participants who did not reply within a reasonable time. The present questionnaire was a compilation from a questionnaire previously used in Norway in 2011 [13], translated into English by native speaker, and back-translated again to assess potential discrepancies. Thereafter, the questionnaire was translated into Icelandic (IBA and TJ) (*the questionnaire is available upon request to the corresponding author*).

**Figure 1:**
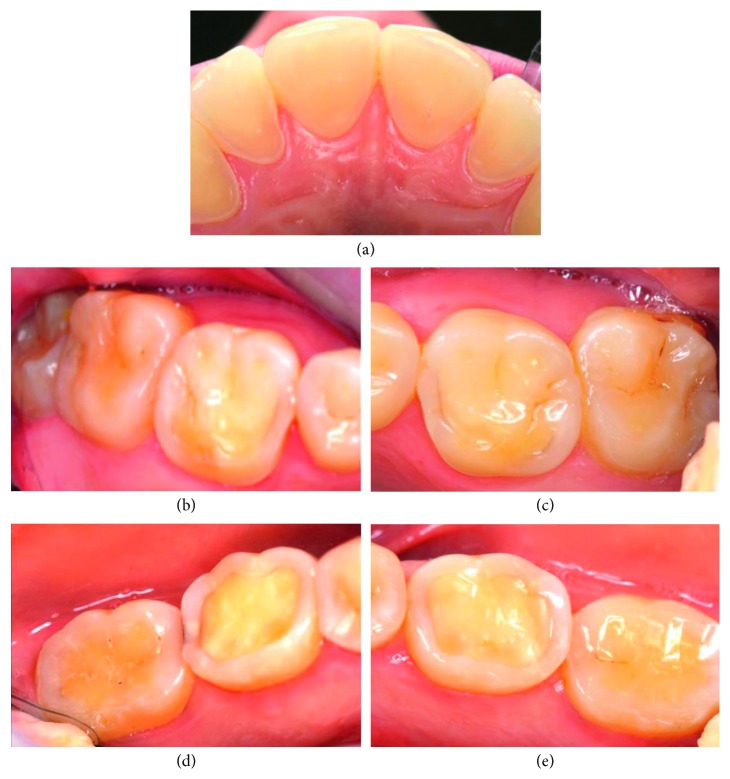
Clinical photographs of patient case 1: a 28-year-old woman who had an eating disorder with vomiting as a teenager, but is now healthy. (a) Palatal surfaces of the upper incisors, (b) occlusal surfaces of upper right 1^st^ and 2^nd^ molars, (c) occlusal surfaces of upper left 1^st^ and 2^nd^ molars, (d) occlusal surfaces of lower right 1^st^ and 2^nd^ molars, and (e) occlusal surfaces of lower left 1^st^ and 2^nd^ molars.

**Figure 2:**
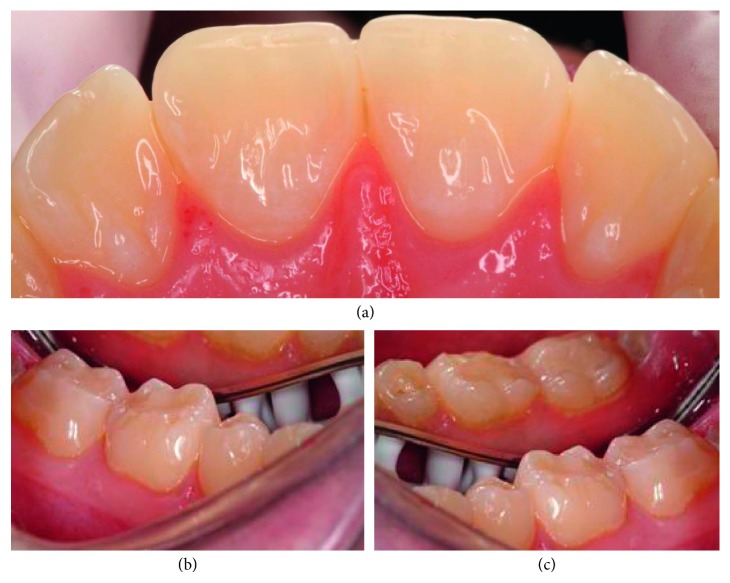
Clinical photographs of patient case 2: a 25-year-old man who suffers from hypersensitivity in the lower molar region. He consumes large amounts of carbonated beverages. (a) Palatal surfaces of the upper incisors, (b) occlusal surfaces of lower right 1^st^ and 2^nd^ molars, and (c) occlusal surfaces of lower left 1^st^ and 2^nd^ molars.

Information was collected on the respondents' sex, age, home county, and type of dental practice and to which extent the respondents were involved in the diagnosis and treatment of dental erosive wear. The questionnaire had two parts. In part one, the dentists were asked how they registered and documented dental erosive wear in their patients aged 18–30 years. They were also asked questions related to their experience with erosive lesions, such as their estimation of the distribution and prevalence of erosive lesions among their patients. The dentists' opinions on probable causes related to erosive lesions were also recorded. The second part of the questionnaire was based on two patient cases, where the dentists were asked to record their choice of treatment including general patient advice and/or type of restoration. A brief patient history as well as colour clinical photographs of labial and palatal surfaces of upper anterior and molar teeth with differing severity of erosive lesions was provided (Figures 1 and 2). The patient cases and questions are described below.

Patient case 1: A 28-year-old woman who had an eating disorder with vomiting as a teenager, but is now healthy.

Patient case 2: A 25-year-old man who suffers from hypersensitivity in the lower molar region. He consumes large amounts of carbonated beverages.

Question one: *What type of advice would you give this patient (You can make more than one choice)?* (1) Information about dietary and drinking habits; (2) information about brushing technique/habits; (3) recommend rinsing with fluoride; (4) recommend rinsing with chlorhexidine; (5) recommend fluoride tablets; (6) recommend specific toothpaste or rinse; (7) refer to specialist, faculty clinic, or other dentists.

Question two: *How would you treat the teeth in the maxillary anterior and posterior regions and the mandibular posterior teeth (You can make more than one choice)?* (1) No treatment; (2) treat locally with fluoride solution (e.g., 2% NaF, Duraphat®, Fluor Protector®); (3) apply bonding material; (4) apply flowable composite; (5) restore with glass ionomer cement; (6) restore with composite filling material, (7) restore with compomer; (8) restore with ceramic laminate/facet/inlay/onlay; (9) restore with crown.

The questions were identical to a questionnaire study conducted among Norwegian dentists in 2011 [13], with the exact same questions and patient case 1. However, patient case 2 was added in the present study.

### 2.1. Statistical Analysis

The data were processed using SPSS version 24.0 (Statistical Package for the Social Sciences; SPSS Inc., Chicago, Ill., USA). Statistical evaluation was carried out by means of descriptive statistics with chi-squared tests. A significance level of 5% was used.

### 2.2. Ethical Considerations

Participation was voluntary and no compensation was given to the respondents. Anonymity was ensured by QuestBack. The study was reported to the National Bioethics Committee of Iceland, who considered the study as not being subject to notification according to law no. 44/2014 (Act on Scientific Research in the Health Sector No. 44/2014).

## 3. Results

All dentists in Iceland (*n*=341) were invited to participate in the study, and 219 dentists responded after four reminders. A response rate of 64.2% was calculated according to Standard Definitions of the American Association for Public Opinion Research [14]. The respondents ranged in age from 25 to 76 years and consisted of 42% females and 58% males. Respondents who stated that they did not work with patients having dental erosive wear (*n*=66) were excluded from further statistical analyses. Therefore, 153 dentists were included in the study.

Most of the respondents (*n*=143, 96%) reported that they registered dental erosive lesions in their patient's charts. Specific qualitative scoring systems were used by 40% of these dentists; 36% used a two-graded scoring system (enamel-dentine) and 4% used a more detailed system. The severity of lesions was described only in words in the patient charts by 61%, while 4% did not report details on their scoring system. Regarding the quantity of erosive lesions, 22% of the respondents stated that they registered affected surfaces, while 12% registered only affected teeth and 1% registered only at individual-level. Clinical photographs were reported as being “often performed” as documented by 15% of the dentists, while 57% stated “sometimes” and 28% stated “never.” The corresponding values for documentation with study models were 4% (often), 54% (sometimes), and 42% (never). The dentists reported that they most often saw erosive lesions on the occlusal surfaces of first mandibular molars (73%), followed by the palatal surfaces in upper anterior teeth (61%), and the occlusal surfaces of first maxillary molars (36%). Gender differences in the prevalence of erosive lesions were reported by 67% of the dentists. Their opinion was that erosive lesions were more common in male patients than in female patients. On the contrary, only 1% of the dentists stated that more females than males had erosive lesions, while 19% of dentists reported no gender difference and 13% were not sure.

A majority of (54%) the experienced dentists (more than 10–15 years of clinical experience (*n*=106)) reported higher prevalence of erosive lesions today compared with 10–15 years ago. Thirty percent did not think that there were more erosive lesions today, while 16% were not sure.

The most common causes for erosive lesions stated by the dentists were: consumption of carbonated beverages (98%), followed by acidic juices (68%), sport drinks (58%), acidic diet (23%), and fruits (9%). Reflux (54%) and eating disorders with vomiting (20%) were also reported as common causes by the dentists. Most of the dentists (74%) reported that they usually found a probable cause of the erosive lesions, 24% occasionally, and only 2% reported that they either “seldom found a probable cause” or “did not know.”

Only 12% of the dentists reported that they “always” recorded a dietary history in patients with erosive lesions, while 38% reported that they “often” recorded a dietary history, 33% only “occasionally” and 17% reported that they “never” recorded dietary history. The type of dietary questionnaire used varied among the dentists; 4% used a precoded questionnaire, 12% asked the patient to record all they consumed during a certain period of time (amount, time of day etc.), while the remaining 83% used different techniques, such as oral interviews and explanatory text in the dental chart.

With regard to the question about connection between erosive lesions and caries activity, half of the dentists (51%) did not think that patients with dental erosive lesions had more caries than those without erosive lesions. However, 22% of the dentists did report higher caries prevalence among patients with erosive lesions. No differences were reported by 13% of the dentists, while 14% were not sure. Salivary flow rate was “always” or “often” measured by 8% of the dentists, 27% only occasionally; while 65% of the dentists reported that they never measured saliva secretion in patients with dental erosive lesions. Hence, more than half of the dentists (55%) did not have an opinion on the salivary flow rate in patients with erosive lesions compared with other patients. About one-third of the respondents (35%) stated that their patients with erosive lesions had a normal amount of saliva. Decreased salivary flow rate among erosion patients were reported by 9% of the dentists, while 1% reported that the erosion patients had higher salivary rate.

Most of the dentists (88%) chose to treat patients with dental erosive wear themselves, while 5% referred such patients to other dentists or specialists dentists/university faculty clinics. The remaining 7% treated many patients themselves, but referred the more severe cases.

The two presented patient cases were assessed by a previously calibrated examiner (AM), who scored the severity of the erosive lesions on surface level based on the clinical photos using a tested scoring system for erosive lesions (VEDE system) [15]. In patient case 1, enamel lesions (grade 2) were registered on central incisors and upper 2^nd^ molars, while dentine lesions (grades 3, 4, and 5) were seen on lateral incisors, all 1^st^ molars and lower 2^nd^ molars, indicating a case with severe erosive lesions (Figure 1). In patient case 2, no erosive lesions were registered on central incisors, while dentine lesions (grades 3, 4, and 5) were seen on lower 1^st^ and 2^nd^ molars, indicating a case with severe erosive lesions (Figure 2). The dentists' treatment choices in both patient cases are presented in Tables 1 and 2.

## 4. Discussion

Interest in dental erosive wear is increasing, and new recommendations of less invasive approaches to the treatment of the condition are emphasized. Therefore, the main objective of the present study was to identify dentists' knowledge, beliefs, and clinical behaviour toward the condition. The present study is the first of its nature to analyze and permit comparison of the knowledge and treatment decisions of dental erosive wear between different countries, as it is conducted in a similar manner as the recent questionnaire study from Norway. The importance of making such intercountry assessments is high.

The questionnaire used in this study was sent to all dentists working in Iceland. However, only dentists working clinically with dental erosive wear were included. As reported in the previous study, measures were taken to ensure a high response rate [13, 16], and an acceptable reply rate of 64.2% was obtained after four reminders. As any questionnaire study, the present study has some limitations. Unfortunately, as the anonymous design of the questionnaire did not allow for collecting any information from the nonresponders, the potential of nonresponse bias cannot be excluded. Furthermore, assessing the effects of related factors based on questionnaires may not provide accurate data as the answers are limited by the respondents' ability to recall. Possible biases related to this type of study have been addressed and discussed previously [17]. The first limitation is response bias subjected to social desirability bias, leading to responses that are “politically” correct according to guidelines or experts' opinions. The second limitation is the nature of nonvalidated self-reports that may not reflect the actual behaviour. However, a relatively recent study has shown that there is a high concordance between questionnaire responses and actual treatment [18].

As the prevalence of dental erosive wear among children and adolescents in permanent teeth is estimated to be on an average of 30% [2] and is found to be almost equally common in the Nordic countries, it is assumed that clinicians working with young subjects are aware and familiar with the condition. Almost all the dentists (96%) reported that they registered erosive lesions; however, very few used a detailed grading system (4%). This finding was similar to results reported among the Norwegian dentists. As highlighted in previous studies, use of a grading system is required to be able to record the presence, severity, and progression of the lesions [15, 19] and is considered as one of the fundamental clinical procedures to record the effect of preventive strategies toward dental erosive wear.

Most of the dentists (54%) who responded to the present questionnaire assumed that the prevalence of dental erosive wear is higher nowadays compared with 10–15 years ago. A recent study from Norway [4] has shown that the prevalence of the condition is almost unchanged compared with data reported 30 years ago. Another study among kindergarten children reported an increase over the last decade from 31.3% to 45.4% [20]. However, it is difficult to determine if the presence and severity of the erosive lesions really increase over time or if the condition is perceived more often due to a growing interest and knowledge.

In the present study, similar to the Norwegian survey, a majority of the dentists reported a gender difference—67% reported that dental erosive lesions were more common in males than in females. This is also supported in many previous prevalence studies [3, 5, 7–9] which have suggested that a higher consumption of acidic beverages among males could be a significant contributor; however, a recent study also revealed that there may be a genetic explanation [21]. In that study, a significant association between variation in enamel-formation genes and a lower susceptibility to dental erosive wear was revealed among female subjects.

A thorough anamnesis and a comprehensive clinical examination form the basis for diagnosis, risk assessment, and treatment decisions of dental erosive wear [12]. Most of the dentists both in Norway [13] and in Iceland (77% and 74%, respectively) usually found a probable cause of the erosive wear; where carbonated beverages and acidic juices were revealed as the most common contributors to the lesions. However, a dietary history was seldom recorded and only a few dentists used a precoded questionnaire for this. The low priority of recording dietary habits is a concerning finding as these analyses are of major importance to identify possible aetiological factors and may be indicative of individual's risk assessment. Unfortunately, the questionnaire was not designed in a manner to reveal the reason for this low priority; however, one may speculate that clinicians consider it to be time-consuming, that time available does not allow for an extended examination, or that they feel confident in finding the cause in their patients without a dietary questionnaire. After all, most of the dentists (74%) reported that they found a probable cause of the erosive lesions.

One of the surprising findings was that 54% of the dentists from Iceland reported reflux as an important factor leading to dental erosive lesions, while 20% specified eating disorders as a reason. The numbers from Norway identified these conditions as more uncommon causes (8% for both diseases) [13]. The reason for this is not clear, but it may be assumed that focus on reflux and eating disorders as a cause of dental erosion has increased in recent years. It may be anticipated that in Norway, the focus has been on consumption of acidic beverages and other extrinsic factors as the most important causes [22–24], and in Iceland, the interest has been more directed toward intrinsic origin [25, 26]. While Jensdottir et al. found no difference in the prevalence of dental erosion between young Icelandic adults and patients (19–22 years) with gastroesophageal reflux disease (GERD) in 2004 [25], a more recent study from Iceland (2012) found a significant association in 249 individuals (mean age, 22 years) [26]. In Norway (2012), it has been reported that 18-year-olds with a daily or weekly reported reflux symptoms had two times of increased risk for dental erosive wear compared with a monthly or less frequent presence [22]. A systematic review including 17 studies on GERD and dental erosion also showed an association between the two conditions with a median prevalence of erosive lesions in GERD patients of 24% and the median prevalence of GERD in adults with dental erosion of 32.5% [27]. Johansson et al. [28] examined the oral health of patients with eating disorders and compared them to gender- and age-matched controls. Patients with eating disorders were at 8.5 times higher risk of having dental erosion, and those patients with a longer history of eating disorder had more commonly erosive lesions. Similar findings were also reported in a recent study among Norwegian adults suffering from self-induced vomiting [29] and among the adolescents with self-reported vomiting [22]. In the latter study, adolescents with eating disorders had a 1.9 times increased risk of having erosive lesions, and girls were at a higher risk. However, one should bear in mind that the reported presence of vomiting and reflux may be underestimated. Adolescents may fail to report vomiting, as eating disorders often are related to guilt, shame, and self-denial [28]. Studies have also shown that there are patients with reflux oesophagitis who do not show reflux discomfort, known as “silent reflux” [28, 30].

Since dental health personnel often are the first healthcare professionals to whom persons with previously undiagnosed reflux and eating disorders may present [31] and dental care is an important part of the overall treatment for these patients, it is of great importance that dentists have an adequate knowledge about such conditions and how to prevent and treat the oral consequences.

The questionnaire survey from Norway [13] revealed that little or no priority was given to salivary analyses in patients diagnosed with dental erosive wear: the majority did not measure saliva secretion, and one-third did not know the saliva status of their dental erosion patients. Similar results were also reported from the Icelandic colleagues: 65% of the dentists never measured saliva secretion in their patients with dental erosive disease, while 55% had no opinion on the salivary condition in these patients. It is recognized that a high salivary flow rate favours the prevention or minimization of initial acid attack due to the increase in the organic and inorganic constituents of saliva [32]. These components function as buffers and help to maintain the integrity of the teeth [33]. The buffering capacity of saliva is important for prevention and reduction of acidic influence [34], and it has been demonstrated that buffering capacity of the saliva is positively correlated with the secretion rate [35]. Jarvinen et al. [36] found that a low unstimulated salivary flow rate of ≤1 ml/min gave a five times higher risk of dental erosions. In addition to the salivary secretion, several other salivary mechanisms are important during an erosive challenge: dilution and clearance of an erosive agent, neutralization and buffering, and involvement of pellicle formation [33].

Another important purpose of the present survey was to identify dentists' monitoring and treatment strategies illustrated with two patient cases (Figures 1 and 2). The patient case 1 (Figure 1) was identical with the one included in the Norwegian study [13], which illustrated a patient with a combination of mild enamel and severe dentine lesions. The patient case 2 (Figure 2) illustrated an individual with less severe erosive lesions that in the patient case 1, with unaffected palatal surfaces of upper centrals and laterals, but with dentine erosions on the lower molars, from where the hypersensitivity was reported.

For both patients (Figures 1 and 2), the Icelandic clinicians would most commonly give the patient information about dietary and drinking advices, followed by recommendation on use of fluoride rinse and information about brushing technique (Table 1). However, the advice on the use of fluoride tablets was considered as less important. The same pattern was also detected among Norwegian dentists. The importance of awareness toward dietary and drinking habits in patients with dental erosive lesion is, as mentioned previously, unquestionable, and all patients regardless of the cause of the erosive disease should be enlightened about the influence of the dietary/drinking habits. In addition to these advices, fluoride recommendation is considered as an important preventive strategy against dental erosions. Several *in vitro* and *in situ* studies have shown a potential antierosive effect of conventional fluorides, and mouth rinses containing stannous fluoride have shown an erosion reduction between 18 and 50% [37, 38]. A daily treatment regime that could delay or inhibit the development of erosive wear and thereby lower the need for invasive and expensive restorative treatment is of clinical importance. However, the documentation is still too sparse to draw any conclusions [12].

The majority of the clinicians would give advice about brushing techniques to these patients. Previously, the recommendation was to postpone brushing after an acidic attack as the softened tooth surfaces caused by exposure to acidic products are vulnerable to tooth brushing [39]. However, softened enamel is not remineralized by saliva over short time periods, and the tooth substance will be worn away even in the absence of tooth brushing. Therefore, postponing brushing after acidic attacks is not a useful preventive measure, especially as brushing with fluoride toothpaste is important as a source of fluoride in the prevention of caries [12].

When it comes to the dentists' general choice of treatment/restorative material described in Table 2, most of the dentists reported to treat eroded teeth with fluoride solution or bonding material, which may combat hypersensitivities of affected teeth and protect teeth from further erosive attack. This is also in accordance with the current trend toward a minimally invasive approach when treating erosive lesions. In vitro and in situ studies have shown that resin-based sealants were able to protect erosive/abrasive wear of both enamel and dentine [40, 41], and a clinical study has shown a protection for a period of 6–9 months [42].

Another interesting observation was that the dentists chose to treat more frequently the less affected case 2 than the patient case 1. This surprising finding may be associated with patient's reported hypersensitivity in case 2, as the philosophy is that the restorative management aims to reduce symptoms of pain and dentine hypersensitivity [12]. Perhaps, as mentioned previously, this could also be related to dentists' focus on the tooth-level rather than considering the whole individual.

Another worrying finding is that more than 17% of the dentists suggested restoring incisors in patient case 2, which were considered as unaffected, with more invasive options such as veneers, inlays/onlays, or crowns. The reason for that is not clear, but dentists report difficulties to differentiate between the severities of the erosive wear based on the clinical pictures [43]. Even though some dentists wanted to restore teeth more invasively, most of them considered a restoration with a filling. Nowadays, it is a general agreement that the improvements of the composite materials make them suitable for restoration of worn dentitions [44–46], and that the erosive conditions may have only little impact on tooth-coloured restorations [44].

Another interesting observation was that the Norwegian dentist treated operatively affected teeth in case 1 more often [13] than dentists in Iceland and with a more restorative approach. It has been 5 years between the two studies, and one may speculate that the more restrictive treatment may be due to more focus and knowledge on preventive strategies related to management of dental erosive lesions. It has recently been suggested in a consensus report [12] that preventive strategies should be preferred before restorative treatment.

One should bear in mind that at present, there is no standard treatment that can be recommended for teeth with dental erosive wear. Nevertheless, a preventive approach has shown to be clinically successful and should always be recommended.

## 5. Conclusion

Icelandic dentists seem to be well educated for diagnosis and treatment of dental erosive wear and this study suggests that the clinicians in Iceland are relatively updated on this condition. However, little priority was given to documentation, dietary, and salivary analyses. The study suggests that dentists are aware of a minimally invasive approach when treating erosive lesions. It is important to have in mind that when the restorative treatment is indicated of worn dentition, resin composite should be the first material of choice.

## Figures and Tables

**Table 1:** The frequency (%) of dentists' general patient advice in the patient case. *n* = number of dentists responding to each question.

Advice	Patient case 1		Patient case 2	
	*n*	%	*n*	%
Information about good dietary and drinking habits	127	87.6	151	99.3
Recommend rinsing with fluoride	111	76.6	112	73.7
Information about brushing technique/habits	99	68.3	103	67.8
Recommend specific toothpaste or rinse	42	29.0	51	33.6
Refer to specialist, faculty clinic, or other dentist	26	17.9	22	14.5
Recommend fluoride tablets	2	1.4	3	2.0
Recommend rinsing with chlorhexidine	1	0.7	1	0.7

**Table 2:** The frequency (%) of dentists' general choice of treatment and/or type of restorative material in each patient case. *n* = number of dentists responding to each question.

Treatment decision	Patient case 1						Patient case 2			
	Incisors		1^st^ molars		2^nd^ molars		Incisors		1^st^ molars	2^nd^ molars
	Centrals	Laterals	Lower	Upper	Lower	Upper	Centrals	Laterals	Lower	Lower
	*n*=134	*n*=129	*n*=129	*n*=131	*n*=123	*n*=123	*n*=139	*n*=124	*n*=141	*n*=137
No treatment	17.2	17.2	8.5	8.9	9.9	25.2	2.2	5.6	1.4	2.2
Treat locally with fluoride solution or bonding material	61.9	61.9	55.0	56.9	59.5	58.5	63.3	66.1	66.0	65.7
Restore with filling	15.7	15.7	14.0	16.3	17.6	9.8	24.5	21.0	18.4	18.2
Restore with ceramic laminate/facet/inlay/onlay	3.0	3.0	12.4	8.9	6.9	2.4	4.3	2.4	7.1	5.8
Restore with crown	2.2	2.2	10.1	8.9	6.1	4.1	5.8	4.8	7.1	8.0

## Data Availability

The data (original data are in the SPSS version 24.0 (Statistical Package for the Social Sciences; SPSS Inc., Chicago, Ill., USA)) and questionnaire are available upon request to the corresponding author.
